# Risk of cancer in young and older patients with congenital heart disease and the excess risk of cancer by syndromes, organ transplantation and cardiac surgery: Swedish health registry study (1930–2017)

**DOI:** 10.1016/j.lanepe.2022.100407

**Published:** 2022-05-29

**Authors:** Christina Karazisi, Mikael Dellborg, Karin Mellgren, Kok Wai Giang, Kristofer Skoglund, Peter Eriksson, Zacharias Mandalenakis

**Affiliations:** a Institute of Medicine, Department of Molecular and Clinical Medicine, Sahlgrenska Academy, University of Gothenburg, Sweden; b Department of Medicine, Sahlgrenska University Hospital, Diagnosvägen 11, Gothenburg SE-416 50, Sweden; c Adult Congenital Heart Disease Unit, Sahlgrenska University Hospital, Gothenburg, Sweden; d Department of Pediatric Oncology, The Queen Silvia Children's Hospital, Sahlgrenska University Hospital, Gothenburg, Sweden

**Keywords:** Congenital heart disease, Congenital heart defect, Cancer, Thymectomy, Risk factor, Genetics, Transplantation

## Abstract

**Background:**

Increasing survival of patients with congenital heart disease (CHD) will result in an increased risk of age-dependent acquired diseases later in life. We aimed to investigate the risk of cancer in young and older patients with CHD and to evaluate the excess risk of cancer by syndromes, organ transplantation and cardiac surgery.

**Methods:**

Patients with CHD born between 1930 and 2017 were identified using Swedish Health Registers. Each patient with CHD (*n* = 89,542) was matched by sex and birth year with ten controls without CHD (*n* = 890,472) from the Swedish Total Population Register.

**Findings:**

4012 patients with CHD (4·5%) and 35,218 controls (4·0%) developed cancer. The median follow-up time was 58·8 (IQR 42·4–69·0) years. The overall cancer risk was 1·23 times higher (95% confidence interval (CI) 1·19–1·27) in patients with CHD compared with matched controls, and remained significant when patients with syndromes and organ transplant recipients were excluded. The risk of cancer was higher in all CHD age groups, and in patients that underwent cardiac surgery during the first year after birth (Hazard Ratio 1·83; 95% CI 1·32–2·54). The highest risk was found in children (0–17 years), HR 3·21 (95% CI 2·90–3·56).

**Interpretation:**

The cancer risk in patients with CHD was 23% higher than in matched controls without CHD. The highest risk was found in children and in the latest birth cohort (1990–2017).

**Funding:**

Funding by the Swedish state (Grant Number: 236611), the Swedish Research Council (Grant Number: 2019-00193), the Swedish Childhood Cancer Fund (Grant Number: SP2017-0012) and the Swedish Heart-Lung Foundation (Grant Number: 20190724).

**Research in context**

**Evidence before this study**

Patients with congenital heart disease (CHD) are at higher risk of acquired diseases, such as cancer, compared with matched controls without CHD. Some syndromes and transplants are known to increase cancer risk. To our knowledge, no study has confirmed that the cancer risk in CHD patients is still elevated among CHD patients without these syndromes and organ transplants*.* A recent study has shown an association between early thymectomy and future risks of cancer in patients with CHD. We searched PUBMED without date limits, including articles until Dec 1, 2021, using the terms “congenital heart disease”, “heart defects”, “cancer”, “risk factor”, “radiation”, “genetics”, “transplantation” and “thymectomy”. However, convincing data of the underlying risk factors for the acquired cancer risk are scarce.

**Added value of this study**

In this study, the overall cancer risk in patients with CHD was 23% higher compared with matched controls without CHD. Children (0-17 years) from the latest birth cohort (1990-2017) carried the highest risk even when patients with syndromes and organ transplant recipients were excluded. Congenital cardiac surgery was not associated with an increased risk of cancer in the total study population. However, the risk of cancer in children with CHD who underwent cardiac surgery during the first year of life was almost 2-fold higher that of the controls.

**Implications of all the available evidence**

The results of this study confirm the increased cancer risk in patients with CHD. However, the absolute risk is low. Our hope is that this study will highlight the cancer risk in these patients so that clinicians are aware of the risk. Future studies are needed to better understand the underlying causes for this excess risk and evaluate if there is a need for cancer screening in this patient group. Further, our results may indicate that early thymectomy or damage to the thymus gland by sternotomy during cardiac surgery is of great importance. Today, thymectomy is routinely performed in early childhood during surgical correction of CHDs to facilitate surgical access. The results in this study might open up to a discussion about changing the current surgical procedure.

Alt-text: Unlabelled box

## Introduction

Congenital heart disease (CHD) is the most common congenital malformation in newborns and has a prevalence of approximately 9 per 1000 live births.^1^^,^^2^ The survival of patients with CHD has increased continuously during the last 40 years, and more than 97% of children with CHD are expected to reach adulthood.^3^ Increasing survival of patients with CHD will be associated with an increased risk of acquired diseases given the strong influence age has as a risk factor for e.g. atherosclerotic disease as well as neoplastic disease.

The risk of acquired cardiovascular diseases is 10- to 100-fold higher among young patients with CHD compared with matched controls without CHD.^4^, ^5^, ^6^ The association between CHD and other acquired diseases, such as cancer, is of great importance.^7^, ^8^, ^9^ In our recent report, we found that the overall risk of cancer in children and young adult patients with CHD in Sweden was more than twice as high as that of matched controls without CHD,^7^ similar to previous studies.^8^^,^^9^ The cause of this association is likely multifactorial, and radiation exposure 9, 10, 11 and underlying syndromes ^12^^,^^13^ may be possible factors.

For the majority of cases with CHD, the aetiology of the malformation is unknown. However, approximately 10–20% of cases are associated with causes such as chromosomal abnormalities (e.g., Down syndrome, 22q11 deletion syndrome, Edwards syndrome, Patau syndrome, Turner syndrome, Klinefelter syndrome), single mutation in the DNA (e.g., Noonan syndrome), non-syndromal single-gene disorders, or teratogens.^14^^,^^15^ Patients with Down syndrome have a higher risk of CHD and leukemia.^12^ Deletion of 22q11.2 and renin-angiotensin system pathologies may also manifest as both CHD and a predisposition to cancer.^13^ Heart transplantation is an important treatment option for CHD patients with end-stage circulatory failure.^16^ However, organ transplant recipients are at an increased risk of a wide range of malignancies.^17^ Thymectomy is routinely performed in early childhood during surgical correction of CHDs to facilitate surgical access,^18^^,^^19^ and thymectomy could result in an increased risk of cancer.^18^ Cancer screening is an important tool to reduce morbidity and mortality from cancer;^20^ however, patients with CHD have been described as under-screened for malignancies, such as cervical, breast, and colon cancer.^14^^,^^21^

In the present study, we aimed to investigate the risk of developing cancer in both children and adult patients with CHD. We also aimed to evaluate the effect of syndromes and organ transplants in patients with CHD, and the effect of surgery on cancer risk.

## Methods

### Study population and design

This nationwide study was performed using data from the Swedish National Inpatient Register, the Swedish National Outpatient Register, and the Swedish Cause of Death Register (complete since 1968). The Swedish National Inpatient Register was initiated in 1964, with coverage of all cardiothoracic centres from 1970, and with full national coverage since 1987. The Swedish National Outpatient Register has been complete since 2001 and contains data from hospital outpatient visits, from both the public and the private sectors. It is mandatory for all health care providers to report discharge diagnoses to the register, and > 99% of all discharge diagnoses are recorded.^22^ The International Statistical Classification of Diseases and Related Health Problems (ICD) was used to identify all diagnoses.

Patients born between 1930 and 2017 with a diagnosis of CHD were identified through the patient registers and were included in the present study. Each patient with CHD was matched by sex and birth year with ten controls without CHD from the general population, identified from the Swedish Total Population Register. The Swedish National Board of Health and Welfare and Statistics Sweden identified cases and controls and the linkage between the registers. All national registration numbers were replaced with a code in the final dataset by the Swedish National Board of Health and Welfare. The patients were observed from birth until the occurrence of cancer, death, or the end of the study (31 December 2017), with a maximum age of 87 years.

This study was approved by the Gothenburg Regional Research Ethics Board (Gbg 912-16, T 619-18). The tenets of the Declaration of Helsinki were followed, and the need for patient consent was waived because the study was based on anonymised register-based data. This report follows the Strengthening the Reporting of Observational Studies in Epidemiology (STROBE) reporting guidelines.

### Definitions

CHD was defined as present in a patient with at least one registered diagnosis of CHD according to the ICD revisions (ICD-8, ICD-9, or ICD-10) (Supplementary Table 1) in at least one hospital discharge, outpatient visit, or death certificate. The CHD diagnoses were classified in accordance with the hierarchical CHD classification system first described by Botto et al and then modified by Liu et al.^23^^,^^24^ Cancer was defined by ICD codes in accordance with Supplementary Table 2, and syndromes were defined by ICD codes in accordance with Supplementary Table 3. The ICD translation key was used to identify the different ICD codes in ICD-8 and -9.^25^ For some syndromes, the codes in ICD-8 and -9 were very scattered and nonspecific and were excluded. The CHD patients were further divided into those who underwent surgery of the cardiovascular system vs those who did not. Because we wanted to examine the effect of surgical procedures on cancer risk, patients with a cancer diagnosis before a surgical procedure were excluded in this analysis. In order to evaluate the effect of thymectomy for the development of cancer during early childhood, we studied the risk of cancer in children that underwent congenital heart surgery below vs after their first year of life. Surgical procedures of the cardiovascular system were defined according to the classification of operations (Sixth edition, Swedish version) or following the classification of surgical procedures (1.9 edition, Swedish version).

### Statistical analysis

All statistical analyses were performed with R v3.5.2 (R Foundation for Statistical Computing, Vienna, Austria).^26^ For the patients’ baseline characteristics, categorical data are shown as numbers and percentages, while continuous data are presented as mean with standard deviation or median with interquartile range. Follow-up times for both CHD and control populations were estimated from birth until event (cancer), death, or the end of the study period (31 December 2017), whichever occurred first. Incidence rates were reported as per 10,000 person-years and were estimated as the number of events divided by the total follow-up time of the population. Incidence rate ratio (IRR) was defined as the relative difference between CHD and the control population, with 95% confidence intervals (CI). The Fine–Gray method, which handles competing risks, (R package: prodlim) was used to estimate the cumulative incidence. In the present study, death due to all causes other than cancer was considered the competing event. Cox proportional hazard regression models were used to obtain hazard ratios (HR) with 95% CIs, and for all models, the control population was considered the reference population. Additionally, because of the very long-follow-up (non-proportionality for the Cox models), time was divided into intervals of 0–17, 18–39, and 40+ years, and data were analysed separately. After each interval, only those persons who survived, with follow-up time and those who did not have an event (cancer, death or end of study) were included in the next interval. By doing so, we take consideration to mortality before each time interval. All final models met the requirement of proportionality, and a *p*-value of  < 0·05 was considered statistically significant.

Cumulative incidence and Cox hazard ratios can be seen as two complementary approaches. As we see it, the apparent cancer-free survival may be inflated unless adjusted for competing risk of death, a potential error that may increase over time, and in particular over an extended period of follow-up time. In Cox regression analyses, the biological, momentaneous real-life risk of cancer between the two groups are compared. Accordingly, risk of death at any moment will be comparatively minor, in particular among those born 1990 and later, who form the majority (>60%) of the study cohort.

### Role of the funding source

The funders had no role in the design and conduct of the study; collection, management, analysis, and interpretation of the data; preparation, review, or approval of the manuscript; or the decision to submit the manuscript for publication.

## Results

### Baseline characteristics

We identified 89,542 patients born between 1930 and 2017 who were registered with the diagnosis of CHD (44,170 females [49·3%]) and 890,472 controls (436,752 females [49·0%]) who were matched by birth year and sex. The characteristics of our study population are shown in Table 1. The median follow-up time was 58·8 (IQR 42·4–69·0) years for CHD patients and 61·3 (IQR 49·0–69·8) years for controls.

**Table 1:** Characteristics of the study population.

Characteristic	CHD	Controls
All patients, No (%)	89,542 (9·1%)	890,472 (90·9%)
Male	45,372 (50·7%)	453,720 (51·0%)
Female	44,170 (49·3%)	436,752 (49·0%)
Birth cohort		
1930–1949	7026 (7·8%)	70,260 (7·9%)
1950–1969	10,575 (11·8%)	105,750 (11·9%)
1970–1989	17,359 (19·4%)	173,590 (19·5%)
1990–2017	54,582 (61·0%)	540,872 (60·7%)
Age at event*		
Mean, years (SD)	52·6±22·1	57·4±17·6
Median, years (IQR)	58·8 (42·4–69·0)	61·3 (49·0–69·8)

*cancer, death or end of the study period (31 December 2017).

CHD = congenital heart disease. SD = standard deviation. IQR = interquartile range.

### Cancer risk

Of the identified patients, 4012 patients with CHD (4·5%) and 35,218 controls (4·0%) developed cancer. The overall cancer risk was 1·23 (95% CI: 1·19–1·27) in patients with CHD compared with controls. When patients with syndromes and organ transplant recipients were excluded from the study population, the risk decreased to 1·18 (95% CI 1·14–1·22); however, the incidence and risk of cancer remained higher in patients with CHD compared with controls in all age groups and in later birth cohorts (Table 2). The cancer risk was similar for female and male patients with CHD (Table 3).

**Table 2:** Cancer risk in patients with congenital heart disease compared with matched controls, according to age and birth cohort.

	HR (95% CI)		p-value
	All patients	Excluding patients with syndromes and organ transplant recipients	(all vs excluded patients)
Study population	1·23 (1·19–1·27)	1·18 (1·14–1·22)	<0·001 / <0·001
Age group			
0–17 years	3·21 (2·90–3·56)	2·42 (2·15–2·73)	<0·001 / <0·001
18–39 years	1·34 (1·21–1·48)	1·24 (1·12–1·38)	<0·001 / <0·001
40+ years	1·11 (1·07–1·15)	1·11 (1·07–1·15)	<0·001 / <0·001
Birth cohort			
1930–1949	1·03 (0·98–1·08)	1·03 (0·98–1·08)	0·20 / 0·25
1950–1969	1·33 (1·25–1·42)	1·32 (1·24–1·40)	<0·001 / <0·001
1970–1989	1·76 (1·59–1·96)	1·62 (1·45–1·81)	<0·001 / <0·001
1990–2017	2·88 (2·57–3·22)	2·16 (1·89–2·47)	<0·001 / <0·001

HR = hazard ratio. CI = confidence interval.

**Table 3:** Cancer risk in patients with congenital heart disease and matched controls with and without syndromes or organ transplant, according to sex.

	Cancer events in CHD/Total CHD No (%)	Cancer events in controls/ Total controls No (%)	Hazard Ratio (95% CI)
With syndromes and organ transplant - Female - Male	4012 (4·5) / 89,542 (100) 2025 (50·5) 1987 (49·5)	35,218 (4·0)/ 890,472 (100) 18,252 (51·8) 16,966 (48·2)	1·19 (1·14–1·25) 1·27 (1·21–1·33)
Without syndromes or organ transplant - Female - Male	3730 (4·5) / 83,063 (100) 1865 (50·0) 1865 (50·0)	34,937 (3·9) / 888,540 (100) 16,804 (48·1) 18,133 (51·9)	1·18 (1·14–1·22) 1·14 (1·08–1·19) 1·22 (1·16–1·28)

CHD = congenital heart disease. CI = confidence interval.

Because the ICD-8 and -9 diagnostic codes for some syndromes were very scattered and nonspecific, we performed a sensitivity analysis, excluding patients with syndromes and transplant recipients, by identifying only the patients born between 1997 and 2017 (i.e., patients fully recorded according to the diagnostic codes from ICD-10). This analysis showed that the cancer risk remained elevated even when patients with syndromes and organ transplant recipients were excluded: HR for the total population born between 1997 and 2017 was 2·16 (95% CI 1·89–2·47) (Supplementary Table 4).

**Figure 1:**
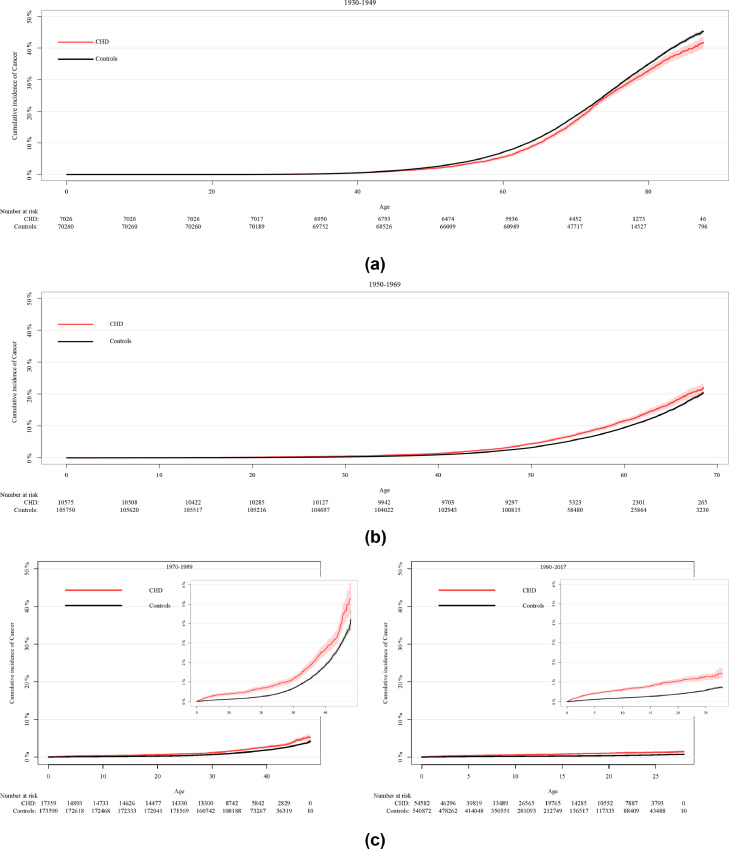
Cumulative incidence of cancer by birth cohort. (a) Birth cohort 1930–1949. (b) Birth cohort 1950–1969. (c) Birth cohorts 1970–1989 and 1990-2017. *CHD = congenital heart disease*.

Figure 1 shows the cumulative incidence of cancer according to birth cohort. The cancer was increased, respectively in cases and controls from the elderly cohorts (Figure 1a and 1b); however, we found the incidence of cancer was significantly higher in patients with CHD from the youngest cohorts (Figure 1c). The youngest patients from all birth cohorts (0–17 years) had the highest HR at 3·21 (95% CI 2·90–3·56), or HR 2·42 (95% CI 2·15–2·73, when patients with syndromes and organ transplant recipients were excluded. HRs were also calculated separately for all patients 0–17 years old in all birth cohorts, and the values were similar (Supplementary Table 5). Furthermore, the cancer risk was higher with younger birth cohorts, with patients born between 1990 and 2017 having the highest HR at 2·88 (95% CI 2·57–3·22), or HR 2·16 (95% CI 1·89–2·47), when patients with syndromes and organ transplant recipients were excluded.

We also calculated the HR for cancer risk for the different lesion groups (Supplementary Table 6). The HR for the entire population was significantly elevated, except for lesion group 2 (severe nonconotruncal defects) and lesion group 5 (atrial septal defect), where results were not statistically significant after excluding patients with syndromes and transplant recipients.

The incidence rate was higher for all types of cancer, for patients with CHD compared with controls, except for cancer of independent (primary) multiple sites (Supplementary Table 7). The most common types of cancer in CHD patients reflected that of the control group, after excluding patients with syndromes and transplant recipients. However, for the entire population, the cancer diagnosis with the highest IRR (e.g., the cancer type with the biggest difference in incidence rate between patients with CHD and their controls) was primary cancer in lymphoid, haematopoietic, or related tissue (IRR 1·89; 95% CI 1·72–2·06). After excluding patients with syndromes and organ transplant recipients, the cancer diagnosis with the highest IRR was cancer of the lip, oral cavity, and pharynx, with an IRR of 1·60 (95% CI 1·32–1·92), followed by primary cancer in lymphoid, haematopoietic, or related tissue (IRR 1·56; 95% CI 1·41–1·72).

The incidence ratio for the different age groups is presented in Supplementary Tables 8–10. In the youngest age group (0–17 years), the most common cancer diagnosis for both patients with CHD and controls was primary cancer in lymphoid, haematopoietic, or related tissue, where the IR for CHD patients was more than twice that of the controls. For the age groups 18–39 years and 40+ years, the most common cancer diagnosis was melanoma and other malignant neoplasms of the skin (including basal cell carcinomas and squamous cell carcinomas of the skin) for both CHD patients and controls.

Congenital cardiac surgery was not associated with an increased risk of cancer. On the contrary, the HR was significantly higher for patients who did not undergo surgery (HR 1·22, 95% CI 1·17–1·27) and lower for patients who underwent surgery (HR 0·84, 95% CI 0·79–0·90). However, children who underwent congenital cardiac surgery during the first year of life, had a higher risk of cancer compared with controls (Supplementary Table 11).

## Discussion

In the present study, we investigated the development of cancer in patients with CHD from birth to a maximum age of 87 years. We found that the cancer risk was 23% higher in patients with CHD compared with matched controls and 18% higher after excluding patients with syndromes and transplant organ recipients. The latest cohort of patients with CHD (0–17 years) had more than twice the cancer risk, particularly of lymphoid or haematopoietic origin, compared with controls. The cancer risk also appeared to increase by birth cohort, and we found the highest risk in patients born between 1990 and 2017. This may reflect an increase in survival during recent decades; however, this may also be because of an increase of cancer, in general. We found a tendency toward an association between later birth cohort and higher cancer risk.

What causes the increased cancer risk in patients with CHD? One of the answers is probably genetic predisposition. Some syndromes are known to have an increased risk of both CHD and cancer.^12^^,^^13^ However, even when we excluded patients with syndromes and organ transplant recipients, who also have a known increased cancer risk,^17^ the risk remained elevated.

Recently, Morton et al. showed that loss-of-function variants in cancer risk genes were increased in patients with CHD.^27^ Vecoli et al showed that adult patients with surgically-repaired CHD had significantly shorter leukocyte telomere length compared with control groups. Short telomeres have been reported to be common and prevalent early genetic alterations in the initiation of most cancer types.^28^ Genetic predisposition may also be a reason why the youngest patients (0–17 years) in all birth cohorts appeared to have the highest cancer risk, with a hazard ratio at least two-fold higher than that of matched controls.

There is also an ongoing discussion about the role of low-ionisation radiation and the risk for cancer in CHD patients.^10^^,^^11^^,^^29^ Unfortunately, in this study, we had no data describing the amount of radiation to which the patients were exposed. One can argue that it is likely that patients who have undergone surgery have received more radiation. In our data, when comparing the cancer risk for CHD patients who underwent surgery vs controls and CHD patients who did not undergo surgery vs controls, we did not find a higher cancer risk in patients undergoing surgery. Further studies are necessary to evaluate radiation as a risk factor for cancer in patients with CHD.

In a Swedish nationwide population-based cohort, an association between early thymectomy and future risks of cancer was found in patients with thymectomy compared with age- and sex-matched general population controls.^18^ In the current study, when dividing the patients according to the age of first cardiac surgery, we observed a tendency to a significantly high cancer risk during childhood, particularly in children under one year of age. This could support the hypothesis that very early thymectomy is associated with a higher cancer risk. Unfortunately, in our study, data for the number of repeat congenital cardiac surgeries were not available to study a higher cancer risk for each operation in patients with CHD.

In our study, the cancer diagnosis with the highest IRR was primary cancer in lymphoid, haematopoietic, or related tissue (second highest when excluding patients with syndromes and organ transplant recipients). When we divided the patients into different age groups, primary cancer in lymphoid, haematopoietic, or related tissue was the most common in the youngest age group (0–17 years); third most common in the younger adult group (18–39 years); and sixth most common in the +40 years group. Previous studies have also shown an association between CHD and haematologic cancer.^8^^,^^9^

### Strengths and limitations

Our study has several strengths and limitations. The major strength is that this was a nationwide study that included all patients with a CHD diagnosis in Sweden. Current data suggest that the overall positive predictive value of diagnoses in the Swedish Inpatient Register is high (85%–96%).^22^ In a recent study by Fedchenko et al, CHD diagnosis in the National Swedish Patient Register was validated for patients with both CHD and myocardial infarction diagnoses and showed a positive predictive value for CHD of 74·8%, similar to findings in other studies that have validated other diagnoses in the National Swedish Patient Register.^30^

One of the limitations is that our study involved only registry data, and we did not have access to clinical information, such as radiation exposure. Another limitation is that the Swedish National Inpatient Register was initiated in 1964, with coverage of all cardiothoracic centres from 1970, and with full national coverage since 1987. Therefore, a survivorship bias in patients and controls born before 1970 may be present. Furthermore, The Swedish National Outpatient Register was not complete until 2001.

## Conclusions

In this nationwide, register-based cohort study, we found that the overall cancer risk in patients with CHD was 23% higher compared with matched controls without CHD. The youngest CHD patients (0–17 years) and CHD patients from the latest birth cohort (1990–2017) carried the highest risk even when patients with syndromes and organ transplant recipients were excluded. However, the absolute risk is low. Congenital cardiac surgery was not associated with an increased risk of cancer in the total study population. However, the risk of cancer in children with CHD who underwent cardiac surgery during the first year of life was almost 2-fold higher that of the controls. This may indicate that early thymectomy or damage to the thymus gland by sternotomy during cardiac surgery is of great importance. Further studies are essential to study other risk factors that may be related to cancer in this vulnerable group of patients.

## Funding

Funding by the Swedish state under an agreement between the Swedish Government and County Councils, the ALF-agreement (Grant Number: 236611), the Swedish Childhood Cancer Fund (Grant Number: SP2017-0012), Swedish Research Council (Grant Number: 2019-00193) and the Swedish Heart-Lung Foundation (Grant Number: 20190724).

## Funding/Support

The study was funded by the Swedish state under an agreement between the Swedish Government and County Councils, the ALF-agreement (Grant Number: 236611), the Swedish Research Council (Grant Number: 2019-00193), the Swedish Childhood Cancer Fund (Grant Number: SP2017-0012) and the Swedish Heart-Lung Foundation (Grant Number: 20190724).

## Role of the funder/sponsor

The funders had no role in the design and conduct of the study; collection, management, analysis, and interpretation of the data; preparation, review, or approval of the manuscript; and the decision to submit the manuscript for publication.

## Additional information

The raw data for our estimates are potentially identifiable, and access to the data is restricted. Researchers wishing to access the individual-level data must apply for permission through a research ethics board and from the primary data owners, the Swedish National Board of Health and Welfare and Statistics Sweden.

## Contributors

CK, MD, KM, KWG, KS, PE and ZM contributed to the conception and design of the study. CK, ZM and KWG did the experimental design and the analyses. CK and ZM prepared the manuscript and verified the data. CK, MD, KM, KWG, KS, PE and ZM critically reviewed the manuscript and approved the final manuscript. All authors had full access to all the data in the study and had final responsibility for the decision to submit for publication.

## Declaration of interests

None reported.

## Appendix Supplementary materials

Image, application 1
