# Corrigendum to “Risk of cancer in young and older patients with congenital heart disease and the excess risk of cancer by syndromes, organ transplantation and cardiac surgery: Swedish health registry study (1930–2017)” [The Lancet Regional Health–Europe 18 (2022) 100407]

**DOI:** 10.1016/j.lanepe.2024.100911

**Published:** 2024-04-16

**Authors:** Christina Karazisi, Mikael Dellborg, Karin Mellgren, Kok Wai Giang, Kristofer Skoglund, Peter Eriksson, Zacharias Mandalenakis

**Affiliations:** a Institute of Medicine, Department of Molecular and Clinical Medicine, Sahlgrenska Academy, University of Gothenburg, Sweden; b Department of Medicine, Sahlgrenska University Hospital, Diagnosvägen 11, Gothenburg SE-416 50, Sweden; c Adult Congenital Heart Disease Unit, Sahlgrenska University Hospital, Gothenburg, Sweden; d Department of Pediatric Oncology, The Queen Silvia Children's Hospital, Sahlgrenska University Hospital, Gothenburg, Sweden

The authors have noticed an error in the Supplementary Material Table 1, where accidentally a list in progress instead of the final list, used for the analysis, have initially been published. The corrected list (used for the analysis) can be found below:

**Supplementary Table 1:** Congenital heart disease diagnosis according to the international statistical classification of diseases and related health problems.

Diagnosis	ICD-8	ICD-9^a^	ICD-10
Tetralogy of Fallot	746.29	745C	Q21.3
Transposition of the great vessels	746.19	745B	Q20.3
Common arterial trunk	746.0	745A	Q20.0
Ventricular septal defect	746.39	745E	Q21.0
Atrial septal defect or patent foramen ovale	746.42	745F	Q21.1
Congenital tricuspid stenosis or atresia	746.54	746B	Q22.4
Ebstein’s anomaly	746.54	746C	Q22.5
Congenital stenosis of the aortic valve	746.73	746D	Q23.0
Congenital insufficiency of the aortic valve	746.79	746E	Q23.1
Congenital mitral stenosis	746.59	746F	Q23.2
Congenital mitral insufficiency	746.59	746G	Q23.3
Hypoplastic left heart syndrome	746.74	746H	Q23.4
Congenital subaortic stenosis	746.79	746W	Q24.4
Cor triatriatum	746.89	746W	Q24.2
Infundibular pulmonic stenosis	746.63	746W	Q24.3
Congenital coronary artery anomalies	747.69	746W	Q24.5
Congenital heart block	746.89	746W	Q24.6
Coarctation of the aorta	747.19	747B	Q25.1
Interruption of the aortic arch	747.19	747B	Q25.2, Q25.3
Other unspecified congenital malformations of the aorta	747.29	747C	Q25.4, Q25.8, Q25.9
Congenital malformations of the pulmonary artery	747.34, 747.39	747D	Q25.5–Q25.7
Congenital malformations of the great veins	747.49, 747.59	747E	Q26.0-Q26.4
Cor biloculare	746.89	745H	Q20.8
Double outlet right ventricle	746.19	745B	Q20.1
Double outlet left ventricle	746.19	745B	Q20.2
Double inlet ventricle	746.37	745D	Q20.4
Congenitally corrected transposition	746.19	745B	Q20.5
Isomerism of atrial appendages	745.89	745W	Q20.6
Unspecified congenital malformations of the cardiac chambers	746.89	746X	Q20.8, Q20.9
Atrioventricular septal defect	746.47, 746.46, 746.43	745G	Q21.2
Aortopulmonary septum defect	746.09	745W	Q21.4
Other congenital malformations of the cardiac septum	745.89	745W	Q21.8
Unspecified congenital malformations of the cardiac septum	746.99	745X	Q21.9
Pulmonary valve atresia	746.64	746A	Q22.0
Congenital stenosis of the pulmonary valve	746.63	746A	Q22.1
Congenital pulmonary valve insufficiency	746.69	746A	Q22.2
Other congenital malformations of the pulmonary valve	746.69	746A	Q22.3
Hypoplastic right heart syndrome	746.69	746B	Q22.6
Other congenital malformations of the tricuspid valve	746.54	746B	Q22.8, Q22.9
Other congenital malformations of aortic and mitral valves	746.89	746W	Q23.8, Q23.9
Other specified congenital malformations of the heart	746.89	746W	Q24.8
Unspecified congenital malformations of the heart	746.99	746X	Q24.9
Patent ductus arteriosus	747.09	747A	Q25.0

ICD, International Classification of Diseases.

a Swedish version of the ICD-9.

Since this is the list with ICD codes used in the study, this does not change any of the interpretations and conclusions in the paper, but nevertheless of course we regret this. If you have any further questions or comments, we will of course be happy to address them.

