# Supplementary material for: Risk of cancer in young and older patients with congenital heart disease and the excess risk of cancer by syndromes, organ transplantation and cardiac surgery: Swedish health registry study (1930–2017)
Source: Lancet Reg Health Eur. 2022 May 29;18:100407. doi: 10.1016/j.lanepe.2022.100407 (PMC9156800; doi:10.1016/j.lanepe.2022.100407)
Supplement: Supplementary file 1 [file mmc1.docx]

**Supplementary material**

**Table of contents Page**

**Supplementary Table 1.** Congenital heart disease diagnosis according to the International 2-3
Statistical Classification of Diseases and Related Health Problems

**Supplementary Table 2.** Cancer diagnosis according to the International Statistical 4
Classification of Diseases and Related Health Problems

**Supplementary Table 3.** Syndromes and transplant recipients’ diagnosis according to the 5
International Statistical Classification of Diseases and Related Health Problems

**Supplementary Table 4.** Study population 1997-2017 **–** Characteristics and risk of cancer 5
in patients with congenital heart disease and matched controls

**Supplementary Table 5**. Risk of cancer in patients with congenital heart disease and 6
matched controls according to birth cohort and age groups (HR, 95% CI).

**Supplementary Table 6:** Cancer risk in patients with congenital heart disease and matched 6
controls according to lesion group

**Supplementary Table 7.** Cancer diagnosis and incidence rate of cancer in patients with 7
CHD and controls (*numbers in italic: population without syndromes and organ transplants*)

**Supplementary Table 8.** Cancer diagnosis and incidence rate of cancer in patients with 8
CHD and controls (*numbers in italic: population without syndromes and organ transplants*)
– age 0-17 years

**Supplementary Table 9.** Cancer diagnosis and incidence rate of cancer in patients with 9
CHD and controls (*numbers in italic: population without syndromes and organ transplants*)
– age 18-39 years

**Supplementary Table 10.** Cancer diagnosis and incidence rate of cancer in patients with 10
CHD and controls (*numbers in italic: population without syndromes and organ transplants*)
– age 40+ years

**Supplementary table 11:** Cancer risk in patients with congenital heart disease who did or 11
did not undergo congenital cardiac surgery compared with matched controls (Patients with
syndromes, transplant recipients, and patients with prior cancer before their surgery were
excluded.)

**Supplementary Table 1. Congenital heart disease diagnosis according to the International Statistical Classification of Diseases and Related Health Problems**

| **Diagnosis** | **ICD-8** | **ICD-9*** | **ICD-10** |
| --- | --- | --- | --- |
| Tetralogy of Fallot | 746.29 | 745C | Q21.3 |
| Transposition of the great vessels | 746.1 | 745B | Q20.3 |
| Common arterial trunk | 746.0 | 745A | Q20.0 |
| Ventricular septal defect | 746.39 | 745E | Q21.0 |
| Atrial septal defect or patent foramen ovale | 746.4 | 745F | Q21.1 |
| Congenital tricuspid stenosis or atresia | 746.54 | 746B | Q22.4 |
| Ebstein’s anomaly | 746.54 | 746C | Q22.5 |
| Congenital stenosis of the aortic valve | 746.73 | 746D | Q23.0 |
| Congenital insufficiency of the aortic valve | 746.79 | 746E | Q23.1 |
| Congenital mitral stenosis | 746.59 | 746F | Q23.2 |
| Congenital mitral insufficiency | 746.59 | 746G | Q23.3 |
| Hypoplastic left heart syndrome | 746.74 | 746H | Q23.4 |
| Congenital subaortic stenosis | 746.79 | 746W | Q24.4 |
| Cor triatriatum | 746.82 | 746W | Q24.2 |
| Infundibular pulmonic stenosis | 746.63 | 746W | Q24.3 |
| Congenital coronary vessel anomalies | 746.85 | 746W | Q24.5 |
| Congenital heart block | 746.86 | 746W | Q24.6 |
| Coarctation of the aorta | 747.19 | 747B | Q25.1 |
| Interruption of the aortic arch  (atresia or stenosis of the aorta) | 747.19 | 747B | Q25.2, Q25.3 |
| Other unspecified congenital malformations of the aorta | 747.29 | 747C | Q25.4, Q25.8, Q25.9 |
| Congenital malformations of the pulmonary artery | 747.34, 747.39 | 747D | Q25.5–Q25.7 |
| Congenital malformations of the great veins | 747.49, 747.59 | 747E | Q26 |
| Cor biloculare | 746.89 | 745H | Q20.8 |
| Double outlet right ventricle | 746.19 | 745B | Q20.1 |
| Double outlet left ventricle | 746.19 | 745B | Q20.2 |
| Double inlet ventricle | 746.37 | 745D | Q20.4 |
| Discordant atrioventricular connection | 746.19 | 745B | Q20.5 |
| Isomerism of atrial appendages | 745.89 | 745W | Q20.6 |
| Unspecified congenital malformations of the cardiac chambers | 746.89 | 746X | Q20.8, Q20.9 |
| Atrioventricular septal defect | 746.47 | 745G | Q21.2 |
| Aortopulmonary septum defect | 746.09 | 745W | Q21.4 |
| Other congenital malformations of the cardiac septum | 745.89 | 745W | Q21.8 |
| Unspecified congenital malformations of the cardiac septum | 745.99 | 745X | Q21.9 |
| Pulmonary valve atresia | 746.64 | 746A | Q22.0 |
| Congenital stenosis of the pulmonary valve | 746.63 | 746A | Q22.1 |
| Congenital pulmonary valve insufficiency | 746.69 | 746A | Q22.2 |
| Other congenital malformations of the pulmonary valve | 746.00 | 746A | Q22.3 |
| Hypoplastic right heart syndrome | 746.69 | 746B | Q22.6 |
| Other congenital malformations of the tricuspid valve | 746.54 | 746B | Q22.8, Q22.9 |
| Other congenital malformations of aortic and mitral valves | 746.89 | 746W | Q23.8, Q23.9 |
| Congenital phlebectasia | 747.89 | 747G | Q27.4 |
| Other specified congenital malformations of the heart | 746.89 | 746W | Q24.8 |
| Unspecified congenital malformations of the heart | 746.84 | 746X | Q24.9 |
| Patent ductus arteriosus | 747.0 | 747A | Q25.0 |
| Unspecified congenital malformations of the circulation | 747.9 | 747X | Q28.9 |
| Sequestration of the lungs | 748.5 | 748F | Q33.2 |
| Secondary hypertension | 405 | 405 | I15.8, I15.9 |
| Vitium organicum cordis (VOC) | - | - | I33–37 |
| ICD, International Classification of Diseases. *Swedish version of the ICD-9. | | | |

**Supplementary Table 2. Cancer diagnosis according to the International Statistical Classification of Diseases and Related Health Problems**

| **Diagnosis** | **ICD-8** | **ICD-9*** | **ICD-10** |
| --- | --- | --- | --- |
| Malignant neoplasms of lip, oral cavity and pharynx | 140-149 | 140-149 | C00-C14 |
| Malignant neoplasms of digestive organs | 150-159 | 150-159 | C15-C26 |
| Malignant neoplasms of respiratory and intrathoracic organs | 160-163 | 160-165 | C30-C39 |
| Malignant neoplasms of bone and articular cartilage | 170-171 | 170 | C40-C41 |
| Melanoma and other malignant neoplasms of skin | 172-173 | 172-173 | C43-C44 |
| Malignant neoplasms of mesothelial and soft tissue | - | 158,162,163,171,173 | C45-C49 |
| Malignant neoplasms of breast | 174 | 174-175 | C50 |
| Malignant neoplasms of female genital organs | 180-184 | 179-184,236C | C51-C58 |
| Malignant neoplasms of male genital organs | 185-187 | 185-187 | C60-C63 |
| Malignant neoplasms of urinary tract | 188-189 | 188-189 | C64-C68 |
| Malignant neoplasms of eye, brain and  other parts of central nervous system | 190-192 | 190-192 | C69-C72 |
| Malignant neoplasms of thyroid  and other endocrine glands | 193-194 | 193-194 | C73-C75 |
| Malignant neoplasms o ill-defined,  secondary and unspecified sites | 195-198 | 195-199 | C76-C80 |
| Malignant neoplasms, stated or presumed to be primary,  of lymphoid, haematopoietic and related tissue | 200-209 | 200-209 | C81-C96 |
| Malignant neoplasms of independent (primary) multiple sites | 199 | 199 | C97 |
| ICD, International Classification of Diseases. *Swedish version of the ICD-9. | | | |

**Supplementary Table 3. Syndromes and transplant recipients’ diagnosis according to the International Statistical Classification of Diseases and Related Health Problems**

| **Diagnosis** | **ICD8** | **ICD9** | **ICD10** |
| --- | --- | --- | --- |
| Downs syndrom | 7593 | 758A | Q90 |
| Noonan |  |  | Q87.1 |
| Di Georges syndrome (22q11-deletion) |  | 279L | D82.1 |
| Klinefelter | 759,51 | 758H | Q98 |
| Turners | 759,50 | 758G | Q96 |
| Williams-Buren |  |  | Q93.8 |
| Edwards (trisomi 18) | 759,40 | 758C | Q91 |
| Patau (trisomi 13) | 759,41 | 758B | Q91 |
| CFC (cardio-facio-cutant syndrom, |  |  | Q87.8 |
| costellossyndrom, CHARGE, |  |  |  |
| Smith-Magenis |  |  |  |
| Neurofibromatosis type 1 | 743,40 | 237H | Q85.0 |
| Constitutional aplastic anemia | 284 | 284A | D61.0 |
| (incl Fanconis anemia) |  |  |  |
| Heterotaxia (situs inversus) | 759.0 | 759D | Q89.3 |

**Supplementary Table 4. Study population 1997-2017 – Characteristics and risk of cancer in patients with congenital heart disease and matched controls**

| **Characteristics** | **CHD** | **Controls** |
| --- | --- | --- |
| All participants, No | 44,352 (9.2%) | 438,572 (90.8%) |
| Excl. syndrome/transplant recipients | 41,183 (8,6%) | 437,898 (91,4%) |
| Patients with cancer | 248 (0,6%) | 884 (0,2%) |
| Excl. syndrome/transplant recipients | 167 (0,4%) | 844 (0,2%) |
|  |  |  |
| **Hazard ratio  (95% CI)** | |  |
| All participants |  | 2.18 (1.84-2.57) |
| Excl. Syndrome/transplant recipients |  | 2.16 (1.89-2.47) |

**Supplementary Table 5. Risk of cancer in patients with congenital heart disease and matched controls according to birth cohort and age groups (HR, 95% CI).**

| **Birth cohort** | **0-17 years** | **18-39 years** | **40+ years** |
| --- | --- | --- | --- |
| 1930-1949 | *No data available* | 0.82 (0.58- 1.18) p-value 0.29  *0.80 (0.55-1.15)* p-value 0.22* | 1.03 (0.99- 1.08) p-value 0.15  *1.03 (0.99-1.08)* p-value 0.17* |
| 1950-1969 | 2.95 (1.63-5.35) p-value 0.00  *2.43 (1.26-4.69)* p-value 0.01* | 1.42 (1.18-1.70) p-value 0.00  *1.33 (1.10-1.60)* p-value 0.00* | 1.31 (1.22-1.40) p-value 0.00  *1.31 (1.22-1.40)* p-value 0.00* |
| 1970-1989 | 3.26 (2.63-4.04) p-value 0.00  *2.75 (2.15-3.51)* p-value 0.00* | 1.52 (1.33-1.74) p-value 0.00  *1.43 (1,24-1.65)* p-value 0.00* | 1.51 (1.18-1.95) p-value 0.00  *1.48 (1.13-1.92)* p-value 0.00* |
| 1990-2017 | 3.25 (2.88-3.66) p-value 0.00  *2.39 (2.07-2.75)* p-value 0.00* | 1.20 (0.82-1.77) p-value 0.34  *1.16 (0.77-1.74)* p-value 0.49* | *No patients in this age group* |

*without syndromes or organ transplants

**Supplementary Table 6: Cancer risk in patients with congenital heart disease and matched controls according to lesion group**

| **Lesion group** | **HR (95% CI)** | **p-value** |
| --- | --- | --- |
| 1. Conotruncal defects | 1·58 (1·35–1·84) *1·41 (1·20–1·67)** | <0·001 *<0·001* |
| 2. Severe nonconotruncal defects | 1·32 (1·15–1·51) *1·13 (0·97–1·32)** | <0·001 *0*·*12* |
| 3. Coarctation of the aorta | 1·29 (1·13–1·48) *1·21 (1·05–1·40)** | <0·001 *<0*·*001* |
| 4. Ventricular septal defect | 1·26 (1·16–1·38) *1·19 (1·08–1·31)** | <0·001 *<0*·*001* |
| 5. Atrial septal defect | 1·08 (1·02–1·14) *1·04 (0·99–1·10)** | 0·01 *0*·*13* |
| 6. Other heart and circulatory system anomalies | 1·36 (1·29–1·44) *1·33 (1·26–1·41)** | <0·001 *<0·001* |

HR=hazard ratio. CI=confidence interval.

*without syndromes or organ transplants

**Supplementary Table 7. Cancer diagnosis and incidence rate of cancer in patients with CHD and controls (*numbers in italic: population without syndromes and organ transplants*)**

|  | **Cancer diagnosis** | **Events cases/controls** | | **IR*  (cases/controls)** | **IRR (95%CI)** |
| --- | --- | --- | --- | --- | --- |
|  | Melanoma and other malignant neoplasms of skin | | 1119/9634 *1064/9545* | 4.94/3.97 *4.97/3.94* | 1.24 (1.17-1.32) *1.26 (1.18-1.34)* |
|  | Malignant neoplasms of ill-defined, secondary and unspecified sites | | 854/9301 *833/9249* | 3.76/3.82 *3.88/3.81* | 0.98 (0.92-1.05) *1.02 (0.95-1.09)* |
|  | Malignant neoplasms of digestive organs | | 623/5790 *596/5741* | 2.74/2.38 *2.78/2.37* | 1.15 (1.06-1.25) *1.17 (1.08-1.28)* |
|  | Malignant neoplasms of breast | | 529/5187 *522/5176* | 2.33/2.14 *2.44/2.14* | 1.09 (1.00-1.19) *1.14 (1.04-1.25)* |
|  | Malignant neoplasms of male genital organs | | 526/5130 *513/5110* | 2.32/2.11 *2.39/2.11* | 1.10 (1.00-1.20) *1.14 (1.04-1.24)* |
|  | Malignant neoplasms, stated or presumed to be primary, of lymphoid, haematopoietic and related tissue | | 588/3341 *453/3289* | 2.59/1.37 *2.11/1.36* | 1.89 (1.73-2.06) *1.56 (1.41-1.72)* |
|  | Malignant neoplasms of respiratory and intrathoracic organs | | 272/2903 *267/2891* | 1.20/1.19 *1.24/1.19* | 1.00 (0.88-1.14) *1.05 (0.92-1.18)* |
|  | Malignant neoplasms of female genital organs | | 261/2582 *255/2573* | 1.15/1.06 *1.19/1.06* | 1.08 (0.95-1.23) *1.12 (0.98-1.27)* |
|  | Malignant neoplasms of urinary tract | | 259/2302 *245/2283* | 1.14/0,95 *1.14/0.94* | 1.21 (1.06-1.37) *1.21 (1.06-1.38)* |
|  | Malignant neoplasms of eye, brain and other parts of central nervous system | | 193/1509 *163/1458* | 0.85/0.62 *0.76/0.60* | 1.37 (1.18-1.59) *1.27 (1.07-1.48)* |
|  | Malignant neoplasms of mesothelial and soft tissue | | 137/1289 *128/1262* | 0.60/0,53 *0.60/0.52* | 1.14 (0.95-1,35) *1.15 (0.95-1.37)* |
|  | Malignant neoplasms of lip, oral cavity and pharynx | | 135/904 *127/896* | 0.59/0,37 *0.59/0.37* | 1.60 (1.32-1.92) *1.60 (1.32-1.92)* |
|  | Malignant neoplasms of thyroid and other endocrine glands | | 91/603 *82/598* | 0.40/0.25 *0.38/0.25* | 1.62 (1.29-2.01) *1.55 (1.22-1.94)* |
|  | Malignant neoplasms of bone and articular cartilage | | 39/325 *36/321* | 0.17/0,13 *0.17/0.13* | 1.29 (0,91-1.77) *1.27 (0.87-1.77)* |
|  | Malignant neoplasms of independent (primary) multiple sites | | 4/164 *4/162* | 0.02/0,07 *0.02/0.07* | 0.27 (0,08-0,64) *0.29 (0.09-0.68)* |
|  |  | |  |  |  |

* per 10,000 person-years

**Supplementary Table 8. Cancer diagnosis and incidence rate of cancer in patients with CHD and controls (*numbers in italic: population without syndromes and organ transplants*) – age 0-17 years**

| **Cancer diagnosis** | **Events cases/controls** | **IR*  (cases/controls)** |
| --- | --- | --- |
| Malignant neoplasms, stated or presumed to be primary, of lymphoid, haematopoietic and related tissue | 255/618 *142/595* | 2.20/0.50 *1.31/0.48* |
| Malignant neoplasms of eye, brain and other parts of central nervous system | 65/430 *47/393* | 0.56/0.35 *0.43/0.32* |
| Malignant neoplasms of mesothelial and soft tissue | 41/201 *35/191* | 0.35/0.16 *0.32/0.16* |
| Malignant neoplasms of ill-defined, secondary and unspecified sites | 43/200 *34/194* | 0.37/0.16 *0.31/0.16* |
| Malignant neoplasms of urinary tract | 35/91 *30/87* | 0.30/0.07 *0.28/0.07* |
| Malignant neoplasms of lip, oral cavity and pharynx | 31/67 *26/68* | 0.27/0.05 *0.24/0.06* |
| Malignant neoplasms of thyroid and other endocrine glands | 22/94 *19/91* | 0.19/0.08 *0.18/0.07* |
| Malignant neoplasms of digestive organs | 31/67 *18/66* | 0.27/0.05 *0.17/0.05* |
| Malignant neoplasms of respiratory and intrathoracic organs | 18/34 *15/31* | 0.16/0.03 *0.14/0.03* |
| Malignant neoplasms of bone and articular cartilage | 15/81 *14/78* | 0.13/0.07 *0.13/0.06* |
| Melanoma and other malignant neoplasms of skin | 10/62 *8/60* | 0.09/0.05 *0.07/0.05* |
| Malignant neoplasms of male genital organs | 9/29 *8/28* | 0.08/0.02 *0.07/0.02* |
| Malignant neoplasms of female genital organs | 5/30 *4/28* | 0.04/0.02 *0.04/0.02* |
| Malignant neoplasms of breast | 3/14 *3/14* | 0.03/0.01 *0.03/0.01* |
| Malignant neoplasms of independent (primary) multiple sites | 0/4 *0/4* | 0.00/0.00 *0.00/0.00* |
|  |  |  |

* per 10,000 person-years

**Supplementary Table 9. Cancer diagnosis and incidence rate of cancer in patients with CHD and controls (*numbers in italic: population without syndromes and organ transplants*) – age 18-39 years**

| **Cancer diagnosis** | **Events cases/controls** | **IR*  (cases/controls)** |
| --- | --- | --- |
| Melanoma and other malignant neoplasms of skin | 92/801 *81/794* | 1.32/1.05 *1.23/1.04* |
| Malignant neoplasms of ill-defined, secondary and unspecified sites | 60/531 *55/521* | 0.86/0.70 *0.84/0.69* |
| Malignant neoplasms, stated or presumed to be primary, of lymphoid, haematopoietic and related tissue | 66/511 *54/505* | 0.95/0.67 *0.82/0.66* |
| Malignant neoplasms of female genital organs | 54/475 *51/473* | 0.78/0.62 *0.78/0.62* |
| Malignant neoplasms of male genital organs | 51/410 *43/405* | 0.73/0.54 *0.65/0.53* |
| Malignant neoplasms of breast | 39/454 *37/451* | 0.56/0.60 *0.56/0.59* |
| Malignant neoplasms of eye, brain and other parts of central nervous system | 36/274 *29/269* | 0.52/0.36 *0.44/0.35* |
| Malignant neoplasms of digestive organs | 37/258 *29/248* | 0.53/0.34 *0.44/0.33* |
| Malignant neoplasms of thyroid and other endocrine glands | 27/191 *26/191* | 0.39/0.25 *0.40/0.25* |
| Malignant neoplasms of respiratory and intrathoracic organs | 18/95 *18/94* | 0.26/0.12 *0.27/0.12* |
| Malignant neoplasms of lip, oral cavity and pharynx | 16/90 *15/89* | 0.23/0.12 *0.23/0.12* |
| Malignant neoplasms of mesothelial and soft tissue | 14/125 *14/118* | 0.20/0.16 *0.21/0.16* |
| Malignant neoplasms of urinary tract | 14/81 *9/79* | 0.20/0.11 *0.14/0.10* |
| Malignant neoplasms of bone and articular cartilage | 5/57 *4/56* | 0.07/0.07 *0.06/0.07* |
| Malignant neoplasms of independent (primary) multiple sites | 1/14 *1/12* | 0.01/0.02 *0.02/0.02* |
|  |  |  |

* per 10,000 person-years

**Supplementary Table 10. Cancer diagnosis and incidence rate of cancer in patients with CHD and controls (*numbers in italic: population without syndromes and organ transplants*) – age 40+ years**

| **Cancer diagnosis** | **Events cases/controls** | **IR*  (cases/controls)** |
| --- | --- | --- |
| Melanoma and other malignant neoplasms of skin | 1017/8771 *975/8691* | 24.80/20.15 *24.38/20.02* |
| Malignant neoplasms of ill-defined, secondary and unspecified sites | 751/8570 *744/8534* | 18.10/19.51 *18.39/19.48* |
| Malignant neoplasms of digestive organs | 555/5465 *549/5427* | 13.4/12.45 *13.6/12.40* |
| Malignant neoplasms of breast | 487/4719 *482/4711* | 11.82/10.82 *12.00/10.83* |
| Malignant neoplasms of male genital organs | 466/4691 *462/4677* | 11.26/10.71 *11.45/10.70* |
| Malignant neoplasms, stated or presumed to be primary, of lymphoid, haematopoietic and related tissue | 267/2212 *257/2189* | 6.43/5.03 *6.35/4.99* |
| Malignant neoplasms of respiratory and intrathoracic organs | 236/2774 *234/2766* | 5.67/6.30 *5.77/6.30* |
| Malignant neoplasms of urinary tract | 210/2130 *206/2117* | 5.05/4.84 *5.08/4.83* |
| Malignant neoplasms of female genital organs | 202/2077 *200/2072* | 4.87/4.73 *4.95/4.74* |
| Malignant neoplasms of eye, brain and other parts of central nervous system | 92/805 *87/796* | 2.21/1.83 *2.14/1.81* |
| Malignant neoplasms of lip, oral cavity and pharynx | 87/746 *86/739* | 2.09/1.69 *2.12/1.68* |
| Malignant neoplasms of mesothelial and soft tissue | 82/963 *79/953* | 1.97/2.19 *1.95/2.17* |
| Malignant neoplasms of thyroid and other endocrine glands | 42/318 *37/316* | 1.01/0.72 *0.91/0.72* |
| Malignant neoplasms of bone and articular cartilage | 19/187 *18/187* | 0.46/0.42 *0.44/0.43* |
| Malignant neoplasms of independent (primary) multiple sites | 3/146 *3/146* | 0.07/0.33 *0.07/0.33* |

* per 10,000 person-years

**Supplementary table 11: Cancer risk in patients with congenital heart disease who did or did not undergo congenital cardiac surgery compared with matched controls (Patients with syndromes, transplant recipients, and patients with prior cancer before their surgery were excluded.)**

| **Population** |  |  | **HR (95% CI)** | |
| --- | --- | --- | --- | --- |
| **Surgery (all ages)** | |  | 0·84 (0·79–0·90) | |
|  | *Age at first surgery* | |  |  |
|  |  | <1 year | 1·83 (1·32–2·54) | |
|  |  | 1–17 years | 1·38 (1·16–1·65) | |
| **No surgery (all ages)** | | | 1·22 (1·17–1·27) | |

HR=hazard ratio. CI=confidence interval.
